# Compounds that enhance the tailing activity of Moloney murine leukemia virus reverse transcriptase

**DOI:** 10.1038/s41598-017-04765-8

**Published:** 2017-07-26

**Authors:** Yoshiyuki Ohtsubo, Yuji Nagata, Masataka Tsuda

**Affiliations:** Department of Environmental Life Sciences, Graduate School of Life Sciences, Tohoku University, 2-1-1 Katahira, Sendai, 980-8577 Japan

## Abstract

In a previous study, we showed that MMLV-RT has a strong terminal transferase activity, and that the C-, G-, and T-tailing activities are enhanced by dGMP, dCMP, and dAMP, respectively. In this study, to achieve faster reaction and higher tailing efficiency, we screened other compounds for the ability to enhance the tailing activities of MMLV-RT, and determined the corresponding optimal concentrations. The C-, G-, and T-tailing activities were enhanced by guanine, cytosine, and adenine, respectively, and by derivatives thereof, suggesting a transient Watson-Click base pairing between an enhancer molecule and the nucleotide to be incorporated. In the presence of some additives (GMP and GDP for C-tailing and CMP for G-tailing), the tail length increased continuously, resulting in tail lengths of 7 to 15 (GMP and GDP) or 13 to 22 (CMP) nucleotides. Among the compounds that do not induce continuous addition, adenosine, deoxycytidine, and deoxyguanosine mostly enhanced T-, G-, and C-tailings, respectively. The enhancing chemicals described here will improve the feasibility of N-tailing by MMLV-RT in various biotechnological applications.

## Introduction

Moloney murine leukemia virus reverse transcriptase (MMLV-RT)^1^ is widely used for cDNA preparations and cloning^2–5^. In a previous report, we showed that MMLV-RT has a strong activity to append 3′-overhangs to double-stranded DNA ends^6^. The activity is unique in two respects. First, MMLV-RT can append A, C, G, or T, in contrast to known DNA polymerases that append preferentially A-overhangs and append other nucleotides less efficiently^7–10^. Second, the activity is so strong that DNA molecules in a reaction are almost thoroughly appended with an overhang of 1 to 5 nucleotides. The activity is potentially useful because the high tailing efficiency makes it possible to ligate adaptor DNA molecules to every DNA molecule in a reaction, which in turn enables exhaustive analysis of a DNA pool. In addition, the amount of DNA required for analysis is reduced, thereby potentiating single-cell genomics or other analyses of scarce DNA derived from fossil or environmental origins. We also showed that dAMP, dCMP, and dGMP specifically enhanced T-, G-, and C-tailing, respectively. In this study, we sought for additional compounds that enhance the tailing activity of MMLV-RT, and found that adenine, cytosine, and guanine and their related nucleotides enhanced T-, G-, and C-tailings, respectively.

## Materials and Methods

### DNA preparation

FAM70 DNA was prepared as described previously^6^. In brief, a PCR reaction was carried out using a FAM-labeled primer and the product was digested with PvuII. The resulting 70-bp fragment carrying FAM at one end was purified by polyacrylamide gel electrophoresis and ethanol precipitation. The nucleotide sequence of FAM70 is 5′-FAM-AATGATACGGCGACCACCGAGATCTACACTAGATCGCTCGTCGGCAGCGTCAGATGTGTATAAGAGACAG-3′. The concentration of FAM-70 DNA was determined using an Infinite 200 Fluorescence Spectrophotometer (Tecan, Switzerland), and the FAM-labeled primer was used as a standard.

### Tailing reactions

Wild-type MMLV-RT (200 U/µL) was purchased from Nippon Gene (Japan). MMLV-RT (200 U, approximately 1.8 µg of protein) was subjected to SDS-PAGE analysis to confirm its purity; a single band with a size consistent with the expected size (70 kDa) was observed. The reaction mixture contained, in a total volume of 10 µL, 100 fmols of substrate DNA, 50 mM Tris-HCl pH 8.3, 75 mM KCl, 6 mM MgCl_2_, 2 mM DTT, 4 mM dATP, dCTP, dGTP, or dTTP, 4 mM MnCl_2_, and 50 U MMLV-RT. Reactions were carried out in PCR tubes using a thermal cycler (C1000; Bio-Rad, USA) at 30 °C. A and G-tailing reactions proceeded faster than C and T-tailing reactions, and to evaluate the effects of additives, A- and G-tailing reactions were conducted for 2 min and C- and T-tailing reactions for 5 min. An aliquot of 0.5 µL of the reaction mixture was analyzed by capillary sequencing (see below).

### Reagents

Reagents were purchased from Takara (Japan), Sigma-Aldrich (USA), Tokyo Chemical Industry (Japan) or Nacalai Tesuque (Japan). Guanine was dissolved in 0.3 M NaOH, and the other chemicals were dissolved in TE buffer (10 mM Tris-HCl pH 8.0, 1 mM EDTA). Four reagents guanosine (rG), deoxyguanosine (dG), adenosine (rA), and deoxyadenosine (dA) were prepared as saturated solution in TE buffer. The saturated solutions were stored at 4 °C and were heated at 100 °C for 3 minutes and cooled to room temperature (25 °C) before use. The molar extinction coefficients of 24,000, 17,600, 13,300, and 17,200, which were obtained by measuring solutions of authentic rA, dA, rG, and dG, respectively, were used to spectrophotometrically calculate the saturated concentrations.

### DNA length analysis

Changes in size of the FAM70 DNA were analyzed by a capillary sequencing as described previously^6^. Briefly, samples were added to the HiDi formamide-containing GeneScan–500 LIZ Size Standard (Thermo Fisher Scientific, USA), which contains 16 fragments of known size, and size-separated in a 3130xl Genetic Analyzer (Thermo Fisher Scientific, USA). TraceViewer software^6^ was used to analyze the data, in which it aligned different run data by using two peaks from the size standard.

## Results

### Compounds that enhance MMLV-RT tailing reactions

Because C-, G-, and T-tailings are specifically enhanced by dGMP, dCMP, and dAMP, respectively, and transient Watson-Crick base pairing between an enhancer molecule and a deoxyribonucleoside triphosphate molecule about to be incorporated has been proposed^6, 10^, we questioned whether any compound with a base moiety that can base-pair with the base moiety of a nucleoside triphosphate to be incorporated can enhance the tailing reaction. We expected that some such compounds may exhibit enhancing activities superior to those of dGMP, dCMP, and dAMP.

The tailing assay was conducted as described previously^6^, using a 70-bp DNA molecule having one 5′ end labeled with FAM (designated FAM70) and the 3′ end of the FAM-labeled strand being the target for tailing reactions. The FAM-labeled strand was subjected to capillary sequencing, and TraceViewer software was used to analyze the results.

**Figure 1:**
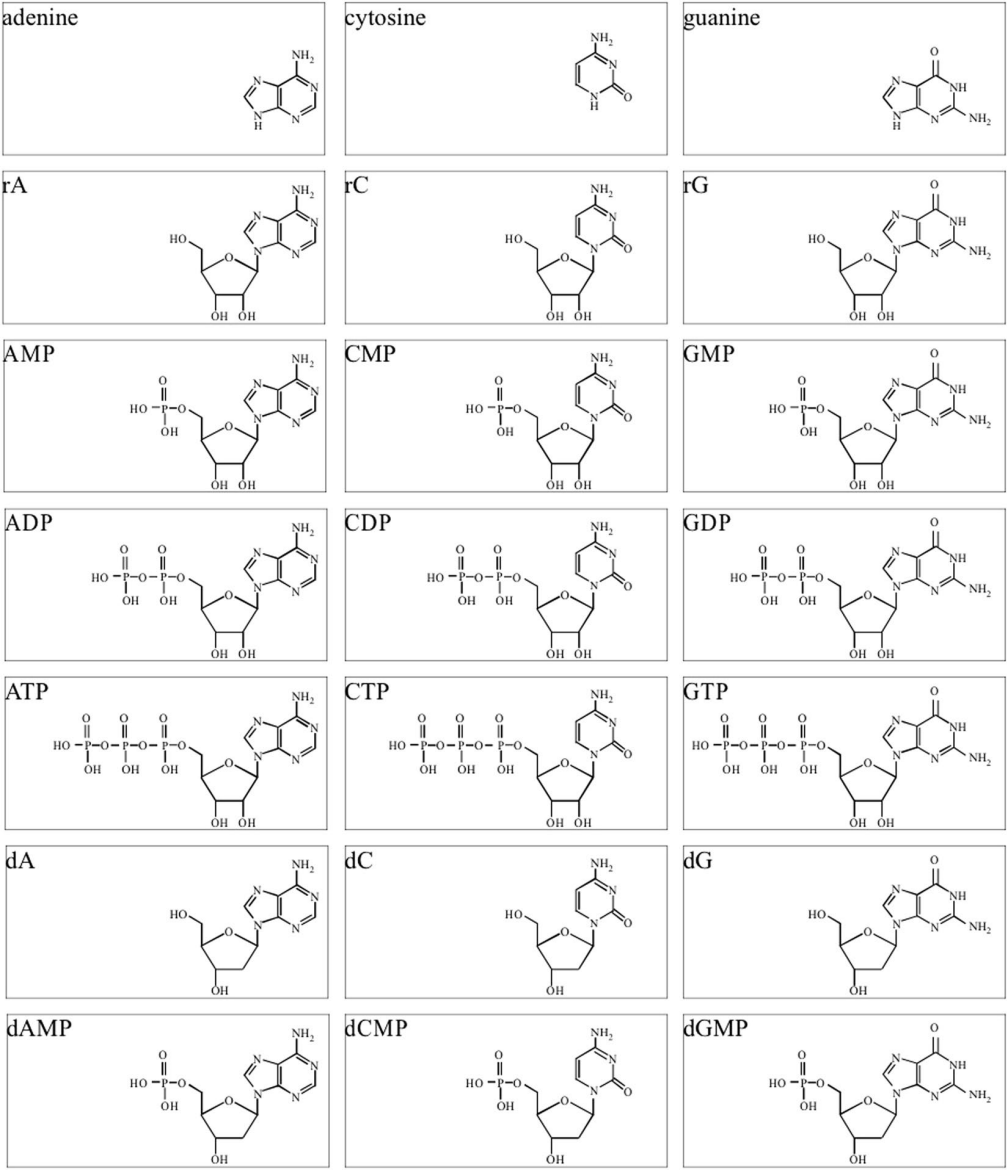
Compounds employed in this study. Abbreviations; rA, adenosine; dA, deoxyadenosine; rC, cytidine; dC, deoxycytidine; rG, guanosine; dG deoxyguanosine.

We investigated the effects of seven types of compounds (see Fig. 1): bases, ribonucleosides, ribonucleoside monophosphates, ribonucleoside diphosphates, ribonucleoside triphosphates (NTPs), deoxyribonucleosides, and deoxyribonucleoside monophosphates. As expected, all seven types of compounds enhanced C-, G-, and T-tailing activities of MMLV-RT (see below). In contrast, 5-methyluridine, thymidine, TMP, thymine, uridine, uridine monophosphate, and UTP, did not enhance A-tailing (data not shown). To date, compounds that enhance A-tailing have not been found.

### Optimal concentrations for tailing reaction enhancement

**Figure 2:**
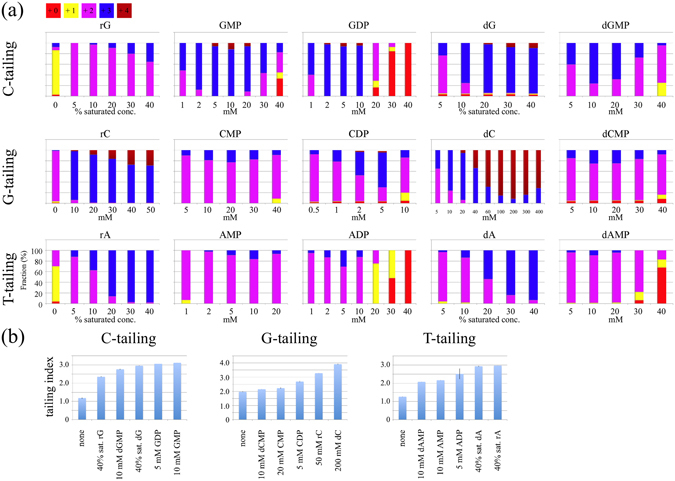
Optimal concentration of compounds that enhance MMLV-RT tailing activity. (**a**) Tailing reactions were conducted in the presence of each compound at different concentrations. Results are shown as averages of triplicate experiments. For dA, rA, dG, and rG, concentrations are shown as the % saturated concentrations, which were measured in TE buffer at 25 °C and were 50 mM, 25 mM, 13 mM, and 2 mM, respectively. (**b**) Tailing index values were calculated from the results at the optimal concentration shown in (**a**).

**Figure 3:**
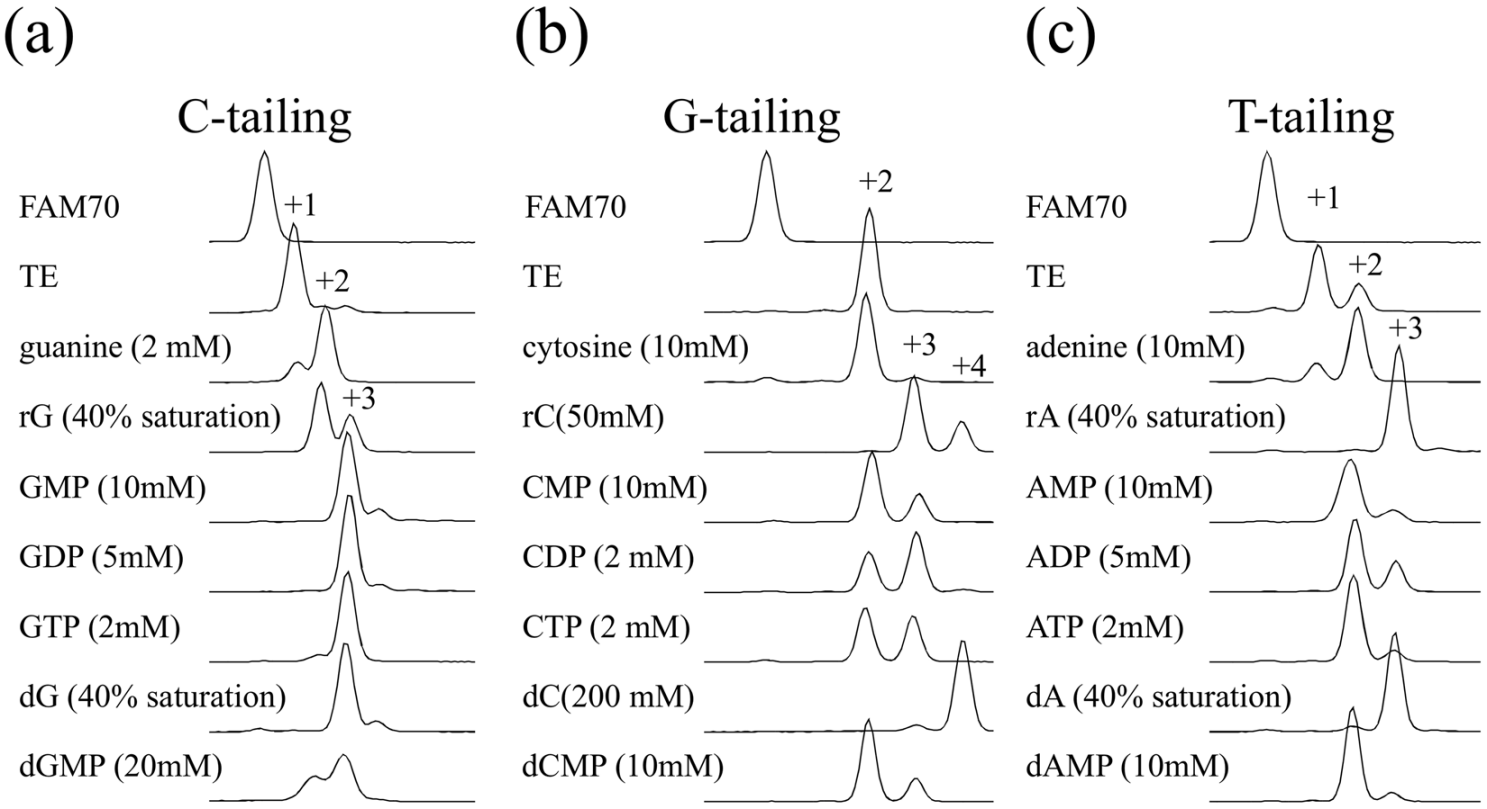
Effects of compounds at their optimum concentration on the tailing activity of MMLV-RT. (**a**) C-tailing, (**b**) G-tailing, and (**c**) T-tailing reactions were conducted in the presence of each compound at its optimal concentration. Some peaks are labeled with the size relative to unreacted FAM70.

**Figure 4:**
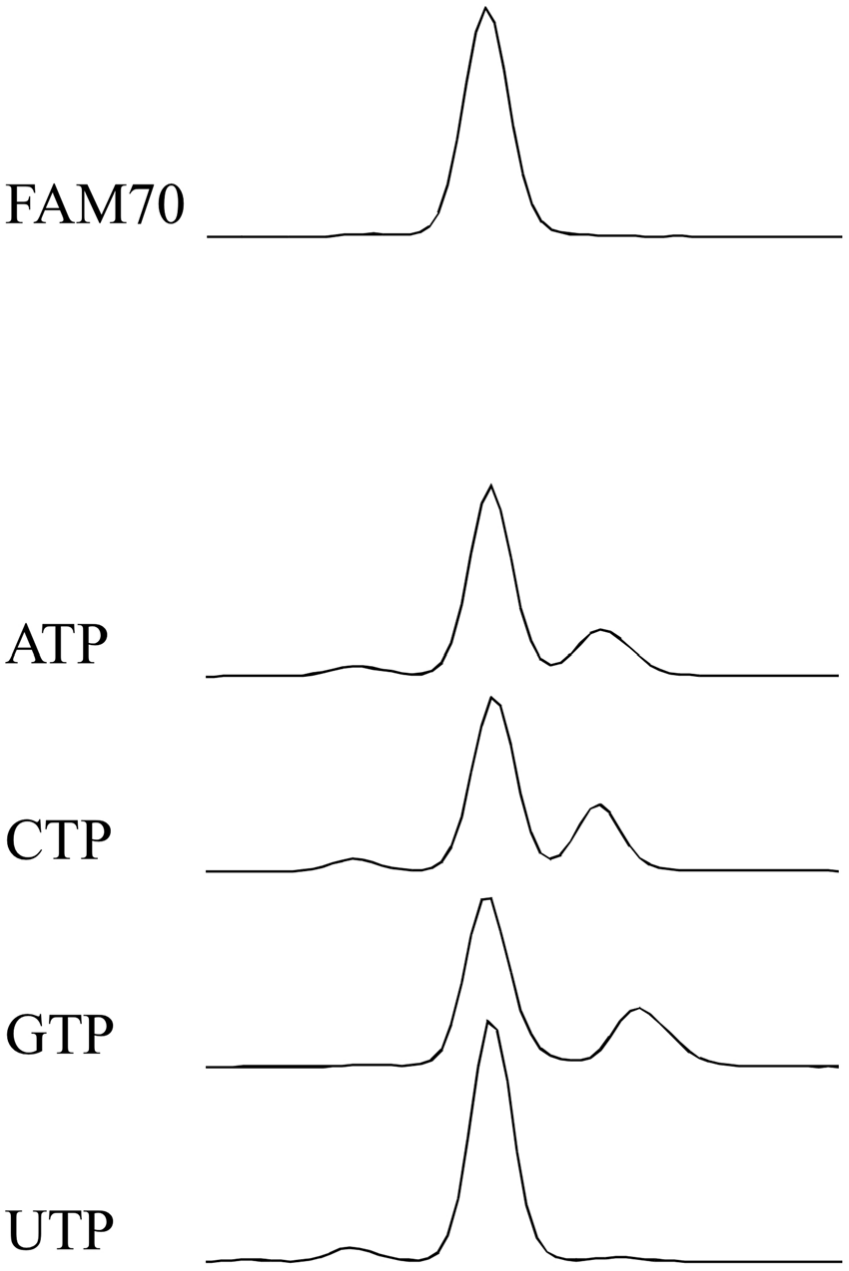
MMLV-RT utilizes ATP, CTP, GTP, and UTP as substrates. FAM70 DNA was incubated with MMLV-RT in the presence of 4 mM MnCl_2_ and 2 mM ATP, CTP, GTP, or UTP for 10 minutes.

We investigated the optimal concentrations of each compound for enhancing the tailing reaction (Fig. 2a), and Fig. 3 shows the results of tailing experiments conducted using compounds at optimal concentrations. We excluded bases, as the enhancing effects were limited and small. We also excluded NTPs because MMLV-RT incorporates NTPs to the ends of double-stranded DNA, although to a limited extent (see Fig. 4). The effects of each compound were evaluated by calculating the tailing index values, which are the average number of nucleotides that a single DNA end has acquired (Fig. 2b). For example, in the absence of additives the G-tailing index was 1.97. In the presence of 10 mM deoxycytidine (dC) the value increased to 2.15, and a concentration of 200 mM dC led to the highest observed G-tailing value of 3.85.

### Continuous tail extension induced by GMP, GDP, and CMP

**Figure 5:**
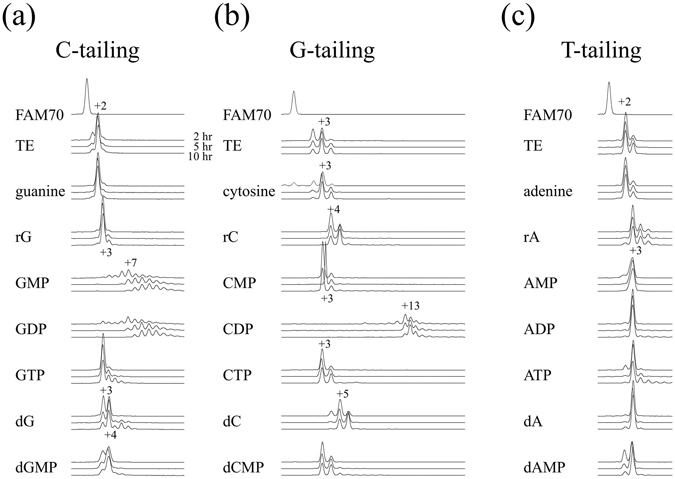
Reaction products after prolonged incubation with MMLV-RT. FAM70 DNA was incubated with MMLV-RT in the presence of each of the enhancing compound for 2 h, 5 h, and 10 h. Concentrations of the compounds are equal to those in Fig. 3. Some peaks are labeled with the size relative to the unreacted FAM70. (**a**) C-tailing, (**b**) G-tailing, (**c**) T-tailing reactions.

We investigated whether the tail is extended continuously or the extension stops after reaching a certain length. Figure 5 shows the tail length profile after 2 h, 5 h, and 10 h of incubation. For C-tailing, GMP and GDP induced continuous addition that led to extensions of 7 to 15 Cs. For G-tailing, CMP induced the continuous addition of Gs (13 to 22).

### Specificities of the tailing reaction enhancements

We investigated the enhancement specificity of compounds that considerably enhanced the tailing reaction, including those that induced continuous extension (Table 1). Most of these compounds enhanced specific tailing reactions, whereas adenosine, which mostly enhanced T-tailing, also enhanced C-tailing. Likewise, CDP enhanced G-tailing and also C-tailing. Some compounds suppressed tailing reactions; dC suppressed A-tailing, GDP suppressed G-tailing, and GMP suppressed G-tailing.

**Table 1:** Effects of additives on A, C, G, and T-tailing^a^.

Additives (concentration)	A-tailing	C-tailing	G-tailing	T-tailing	note
GMP (10 mM)	no effect	++	−	−	induce continuous C-tailing
GDP (5 mM)	−	++	−	−	induce continuous C-tailing
dG (40% sat.)	no effect	++	no effect	no effect	
CDP (2 mM)	no effect	+	+	no effect	induce continuous G-tailing
dC (200 mM)	−	−	+++	no effect	
rA (40% sat.)	no effect	+	no effect	+++	
dA (40% sat.)	no effect	−	no effect	+++	

^a^‘+’, positive effect increasing the tailing index by less than 1; ‘++’, moderate positive effect; ‘+++’ marked positive effect; ‘−’ negative effect.

## Discussion

In this study, the strong tailing activity of MMLV-RT^6^ was further characterized by identifying additional enhancing compounds. The molecular mechanism involved in the tailing enhancement could be the transient Watson-Click base pairing formed between an enhancer molecule and the deoxyribonucleotide triphosphate molecule to be incorporated. We propose that an enhancer molecule binds to the active center of MMLV-RT in an orientation similar to that taken by a single ribonucleotide moiety in an RNA molecule in a revers transcription reaction. Of note, bases are not efficient enhancers, suggesting that the base-moiety and the ribose or deoxyribose moiety of the compounds are involved in the binding. Although we have tested different compounds, other nucleotide analogues, such as cyclic AMP, NADH, and cyclic diGMP, or the wealth of compounds developed for anti-cancer therapy, might have even stronger effects, and the 3D structure of MMLV-RT^11^ and molecular docking approaches may help the search for better tailing enhancers.

Although compounds that are supposed to form Watson-Crick base pairing with dATP were tested, none of them enhanced A-tailing. The base-stacking interaction of dATP with the deoxyribose or base of the DNA ends^7^ might be so strong that the two hydrogen bonds provided by the enhancer molecule do not promote the tailing reaction. In fact, in the absence of enhancer, dATP is incorporated at the fastest rate among dNTPs, and three to five As are added within 10 minutes.

The continuous nucleotide addition by MMLV-RT in the presence of GMP, GDP, and CMP, similar to that of terminal nucleotidyl transferase^12^, might be useful in technological applications, such as labeling of DNA ends, TUNEL assays^13^, RACE^14^, and homopolymer tail-mediated ligation PCR^15^. The different extent of effects of enhancers on the different tailing reactions described in this study will form a basis for utilization of the template-independent tailing activity of MMLV-RT. Although its practical use needs further improvements for each specific application, the activity may be utilized for efficient ligation of adaptor molecules to DNA ends in next-generation sequencing^16–18^, traditional TA^19^ or CG cloning^20^, and cosmid library preparations.
